# Reclassification of genetic-based risk predictions as GWAS data accumulate

**DOI:** 10.1186/s13073-016-0272-5

**Published:** 2016-02-17

**Authors:** Joel Krier, Richard Barfield, Robert C. Green, Peter Kraft

**Affiliations:** Division of Genetics, Department of Medicine, Brigham and Women’s Hospital, Boston, MA USA; Harvard Medical School, Boston, MA USA; Department of Biostatistics, Harvard T.H. Chan School of Public Health, Boston, MA USA; Partners Personalized Medicine, Cambridge, MA USA; Broad Institute, Cambridge, MA USA; Program in Genetic Epidemiology and Statistical Genetics, Harvard T.H. Chan School of Public Health, Boston, MA USA; Department of Epidemiology, Harvard T.H. Chan School of Public Health, Boston, MA USA

## Abstract

**Background:**

Disease risk assessments based on common genetic variation have gained widespread attention and use in recent years. The clinical utility of genetic risk profiles depends on the number and effect size of identified loci, and how stable the predicted risks are as additional loci are discovered. Changes in risk classification for individuals over time would undermine the validity of common genetic variation for risk prediction. In this analysis, we quantified reclassification of genetic risk based on past and anticipated future GWAS data.

**Methods:**

We identified disease-associated SNPs via the NHGRI GWAS catalog and recent large scale genome-wide association study (GWAS). We calculated the genomic risk for a simulated cohort of 100,000 individuals based on a multiplicative odds ratio model using cumulative GWAS-identified SNPs at four time points: 2007, 2009, 2011, and 2013. Individuals were classified as Higher Risk (population adjusted odds >2), Average Risk (between 0.5 and 2), and Lower Risk (<0.5) for each time point and we compared classifications between time points for breast cancer (BrCa), prostate cancer (PrCa), diabetes mellitus type 2 (T2D), and cardiovascular heart disease (CHD). We estimated future reclassification using the anticipated number of undiscovered SNPs.

**Results:**

Risk reclassification occurred for all four phenotypes from 2007 to 2013. During the most recent interval (2011-2013), the degree of risk reclassification ranged from 16.3 % for CHD to 24.4 % for PrCa. Many individuals classified as Higher Risk at earlier time points were subsequently reclassified into a lower risk category. From 2011 to 2013, the degree of such downward risk reclassification ranged from 24.9 % for T2D to 55 % for CHD. The percent of individuals classified as Higher Risk increased as more SNPs were discovered, ranging from an increase of 5 % for CHD to 9 % for PrCa from 2007 to 2013. Reclassification continued to occur when we modeled the discovery of anticipated SNPs based on doubling current sample size.

**Conclusion:**

Risk estimates from common genetic variation show large reclassification rates. Identifying disease-associated SNPs facilitates the clinically relevant task of identifying higher-risk individuals. However, the large amount of reclassification that we demonstrated in individuals initially classified as Higher Risk but later as Average Risk or Lower Risk, suggests that caution is currently warranted in basing clinical decisions on common genetic variation for many complex diseases.

**Electronic supplementary material:**

The online version of this article (doi:10.1186/s13073-016-0272-5) contains supplementary material, which is available to authorized users.

## Background

Risk assessments for common, multifactorial disease based on common genetic variation have long been heralded as a potential clinical application of genomic data [1–6]. The possibility of informative disease risk prediction has gained widespread attention in recent years due to the thousands of disease risk-associated single nucleotide polymorphisms (SNPs) identified from genome-wide association studies (GWAS). Moreover, disease risk prediction based on genetic variation has become increasingly familiar in broader society in part due to the availability and marketing of direct-to-consumer (DTC) genomic testing products [7, 8]. While there are concerns about accurately estimating and communicating common complex risk information [9, 10], customers of DTC genetic testing services discuss their genetic testing results with their physicians and have follow-up testing related to their results [11, 12]. The FDA’s 2013 order to the DTC genomic testing company 23andMe is based in part on the concern that customers may inappropriately use their results to influence their use of medical services [13, 14].

A future role for using personal genetic variation in disease risk assessment continues to be of intense interest and efforts to optimize prediction models for many common medical problems continue. As the technical and cost barriers to whole genome sequencing have decreased significantly in recent years, the application of whole genome sequencing in patients with undiagnosed disorders is growing [15], the sequencing of healthy populations has begun to be explored, and disease risk assessment for common diseases has been demonstrated as a possible application of whole genome sequencing data [16, 17]. In the MedSeq Project, a randomized clinical trial exploring the integration of whole genome sequencing information in clinical care, we are exploring the strengths and limitations of current approaches to risk assessment from common genetic variation, with specific attention to clinical utility and interpretability, and developed a common genetic variant risk assessment for cardiovascular phenotypes [18, 19].

The predictive ability and resulting clinical utility of risk evaluation from common genetic variation depends on the number and effect size of the loci associated with the probability of developing a given phenotype, and has to date been found to generally be modest for most multifactorial conditions. Non-genetic factors such as diet and other exposures will also continue to be important predictors for multifactorial phenotypes such as diabetes. Nevertheless, identifying a subgroup of the population that is at high genetic risk offers clinically relevant opportunities such as enhanced screening or targeted lifestyle modification initiatives.

Many questions remain concerning risk modeling, such as the ability of risk-associated SNPs to capture the genetic architecture for a phenotype and interactions between variants and epigenetic effects [20]. It is reasonable to assume, however, that as additional GWAS are undertaken and overall sample sizes increase, new risk-associated SNPs will be discovered and effect estimates of currently used SNPs will be modified. In addition to enabling novel disease pathway and therapeutic target identification, these SNPs will explain larger proportions of heritability for multifactorial conditions. The genetic component of predictive models will therefore improve and, depending on an individual’s genotype, risk predictions for a given individual will often change. The likelihood of large shifts in risk classification for any given individual as more disease-associated SNPs are identified is not known, however. Moreover, whether the potential for clinically meaningful change in risk assessment diminishes or increases has not been fully explored.

The stability of predicted genetic risks as additional loci are discovered is therefore an important, largely unexplored factor. If genetic risk assessments for a given individual vary significantly over time as new loci are included in the predictive model for a specific condition, it could undermine the validity and utility of common genetic variation for risk prediction that is based upon early SNP discoveries. For example, if an EKG or even a cardiac stress test were ordered for an individual due to an increased genetic risk assessment of coronary heart disease, but subsequent additions of SNPs or effect modifications for existing SNPs in the model caused a reclassification of the individual to a lower risk category, the individual would have undergone unwarranted testing, with the associated unnecessary risk and cost. Conversely, if an individual were originally classified as Lower Risk for a condition and does not pursue standard screening tests or lifestyle modification due to the results, but subsequent identification of high-risk SNPs results in a reclassification to the Higher Risk group, then false reassurance from the initial results might result in missed opportunities for disease risk modification.

A reclassification rate is the percentage of individuals who change predefined risk categories based on change in prediction model or over time. Past published efforts to explore the question of reclassification based on genetic risk predictions include a 2009 study that compared risk reclassification for diabetes mellitus type 2 (T2D) based on one SNP, an 18-SNP model, and the 18-SNP model in addition to non-genetic risk factors and found significant reclassification rates between the three models [21]. We are not aware, however, of updated or more comprehensive evaluations of genetic risk reclassification. The rapid and continuing expansion of the number of statistically significant SNPs in the literature and GWAS catalog offers an opportunity to assess risk reclassification both on recent data and using projected future data based on larger sample sizes. While reclassification rates should eventually diminish as more risk-associated SNPs are discovered, it is not clear if reclassification rates will stabilize, increase or decline in as the next waves of GWAS data are published.

With our primary interest and focus on the near-future clinical applicability of risk prediction from common genetic variation, we analyze reclassification based on past, present, and future GWAS results using the NHGRI GWAS Catalog for four phenotypes [22]. We explore the impact of discovering new SNPs along with modification of effects sizes for four phenotypes: (1) breast cancer (BrCa); (2) prostate cancer (PrCa); (3) diabetes mellitus type 2 (T2D); and (4) coronary heart disease (CHD). We estimate reclassification and model predictive ability for these four phenotypes based on a simulated cohort of 100,000 individuals, first looking backwards at the risk prediction model for each phenotype from GWAS data since 2007. We then explore the impact on reclassification based on a hypothetical future GWAS with double the sample size of the most recent two-stage GWAS for each phenotype.

## Methods

To emulate the methods most widely used by DTC genomic companies and others, our risk prediction model for each phenotype uses SNPs that have been reported at genome-wide significance [10, 16, 23]. Though no best practice has been established for calculating disease risk from multiple risk alleles, we use the multiplicative odds ratio (OR) model in combining the SNPs for each phenotype (equivalent to additive log odds as described in Purcell *et al.*) [10, 19, 24]. To assess how risk prediction could change over time, we account for the continuing discovery of new SNPs and the change in effect size in time for previously identified SNPs.

### SNP identification and filtering

A SNP was considered associated with the disease of interest if the *P* value was less than the genome wide significant cutoff of 5 × 10^-8^. SNPs were identified from the National Human Genome Research Institute (NHGRI) catalog [22] and supplemented by data from four recent large scale studies [25–28]. If one of the recent large-scale studies reported a position for a previously discovered SNP that was different from what was specified in the NHGRI catalog, it was set to the value in the NHGRI catalog. We examined four different diseases: breast cancer (BrCa), prostate cancer (PrCa), T2D, and coronary heart disease (CHD) at four different time points: the end of 2007, 2009, 2011, and 2013. The 2009 risk calculations would therefore include the SNPs that had been reported 2009 or prior.

We considered SNPs within 500 kb of each other to be at the same locus. We included SNPs at a locus in a stepwise fashion, starting with the SNP with the most significant *P* value at a given time point and then adding subsequent SNPs in order of significance if the r^2^ between the candidate SNP and all SNPs already included was below 0.75. Thresholds of 0, 0.25, 0.5, and 1 were also considered to assess the sensitivity of the genetic risk distribution to pruning on different thresholds of LD. We retrieved LD info from the SNP Annotation and Proxy (SNAP) database [29]. Using r^2^ thresholds between 0.25 and 0.75 produced similar results. Using an r^2^ threshold of 1 (that is, include all SNPs at the locus) produced larger genetic risk gradients and area-under-the-curve (AUC) values, but likely overestimates the effect of each locus by double-counting the effect of causal variants tagged by multiple highly correlated SNPs. We adopted this procedure to mimic procedures that rely on catalogs of published GWAS results [17]; more accurate modeling of the contribution of multiple SNPs at a locus can be achieved though conditional or haplotype modeling.

### Simulating cohort

To assess the reclassification in genetic risk values, we estimated the distribution of genetic ORs in cases and in controls. We simulated a cohort of 100,000 individuals of European descent for each of the four conditions evaluated. Assuming the incidence of disease is rare, the distribution of genetic ORs in this cohort approximates the distribution of genetic relative risks in controls. (We repeated the calculations in Tables 3 and 5 explicitly using the distribution in controls for a range of disease incidences from near 0 to 10 %. Results did not appreciably vary from those presented here; differences were mostly under 1 %.) We simulated the genotype for all of the genome-wide significant SNPs discovered from 2007 to 2013, based on the number of SNPs present at each locus. The genotype of each locus was generated independently of other loci. If a locus consisted of one SNP, the genotype was simulated from a binomial distribution using allele frequencies from the 1000 Genomes or HapMap European cohorts, depending upon availability [30, 31]. When a locus contained more than one SNP, a two-fold approach was used. If phased data were available (HapMap or 1000 genomes), the genotype was then bootstrapped from the phased haplotypes for the 100,000 individuals. When phase data were not available, haplotype frequencies were estimated via the EM algorithm [32] and then diplotypes were simulated via a multinomial distribution. Regardless of availability of phase, we removed related individuals using the BioQ notation [33].

To analyze genetic risk values across over time, the genotypic ORs were simulated for SNPs that had been discovered by the four selected dates (2007, 2009, 2011, 2013) and the resulting SNPs were then pruned based on linkage disequilibrium (LD) (see above). The genetic OR for each simulated subject was calculated via a multiplicative model where:

$$ Odd{s}_{i,m}={\displaystyle \prod_{j=1}^{k_m}}O{R}_{j,m}^{g_{i,j}} $$

Here *k*_*m*_ is the number of SNPs known to be associated with a disease at the time point *m*, and *g*_*i,j*_ is the genotype for individual *i* at SNP *j*. If at a later time point a study reported a more significant *P* value for a SNP than had previously been reported, the OR was set to that reported value. The effect size of the SNP is thus allowed to change over time. Thus the OR_j,m_ indicates the OR at time point *m* for SNP *j*. The odds were then normalized by the population mean from each simulated cohort.

$$ {O}_{i,m}=\frac{Odd{s}_{i,m}}{\overline{Odd{s}_i,m}} $$

The normalized OR represents the odds of disease for simulated individual *i* with genotype *g*_*i*_ = (*g*_*i*1_,…,.*g*_*ij*_) relative to the population average odds. While the exact formula used by DTC companies is unique to each company, the mathematical approach used by 23andMe and others is based on some version of the multiplicative odds model used in our study [10].

The cumulative distribution function for genetic ORs in cases is given by:

$$ \Pr \left({O}_{g,m}\le k\right)={\displaystyle {\sum}_g{O}_{g,m} \Pr (g)1\left({O}_{g,m}\le k\right),} $$

and in controls by:

$$ \Pr \left({O}_{g,m}\le k\right)={\displaystyle {\sum}_g \Pr (g)1\left({O}_{g,m}\le k\right),} $$

where Pr(*g*) is estimated using the distribution of genotypes in the simulated cohort. This calculation assumes the disease is rare so that the genetic OR approximates the genetic relative risk.

### Reclassification and AUC

Normalized odds were calculated for the simulated individuals using the known genome-wide significant SNPs known, and their associated ORs for common complex diseases, at four different time points: 2007, 2009, 2011, and 2013. Individuals could be classified at three levels of risk: Lower Risk (O_i_ <0.5); Moderate Risk (0.5 ≤ O_i_ ≤ 2); and Higher Risk (O_i_ > 2) [34, 35]. The percent reclassification was calculated as the percentage of individuals on the off-diagonal of a 3 × 3 table classification table looking between time points. The net reclassification index was calculated as the proportion of cases whose reclassified risk category increased minus the proportion of cases whose reclassified risk category decreased, plus the proportion of controls whose reclassified risk category decreased minus the proportion of controls whose reclassified risk category increased. We chose these cutoffs not because they correspond to any specific clinical decision algorithm, but because they have been used previously as a general benchmark for whether genetic information provides potentially actionable information [35].

The AUC was also calculated at each time point as [36]:

$$ \mathrm{A}\mathrm{U}\mathrm{C}=\upphi \left(\frac{\mathrm{a}}{\sqrt{1+{b}^2}}\right);a=\frac{\mu_D-{\mu}_{\overline{D}}}{\sigma_D};b=\frac{\sigma_{\overline{D}}}{\sigma_D} $$

Where φ is the cumulative distribution for a normal distribution and D represents the case population and $\overline{D}$ represents the control population. The parameters are all in terms of the distribution of the log risks: ${\mu}_{\overline{D}}$ = the mean log genetic OR in controls, ${\sigma}_{\overline{D}}$ = the standard deviation of the log genetic OR in controls, and *μ*_*D*_ and *σ*_*D*_ are the corresponding values for cases.

### Estimating distribution of genetic ORs based on future GWAS

We also estimated the total number of common SNPs that are potentially detectable by GWAS, given a large enough sample size. The number of causal SNPs was calculated via the ‘bin’ method described by Park *et al.* [37], which groups SNPs based on effect size *e* = β^2^2q(1-q). Here β and *q* are the log OR and minor allele frequencies for a SNP, M_j_ is the number of SNPs observed in bin i; p_j_ is the power to observe a SNP with effect size corresponding to bin j, given the largest sample size studied up to the time point [38]:

$$ {M}_{T,j}=\frac{M_j}{p_j};{M}_{Total}={\displaystyle \sum_{j=1}^k}{M}_{T,j} $$

M_T,j_ is thus the total estimated number of SNPs in that bin [37] and *k* is the number of disease-associated SNPs reported in the recent large-scale GWAS studies [25–28, 39]. To simplify power calculations, for this analysis we only used the SNPs reported in recent combined two-stage design studies [25–28, 39]. We are assuming in these calculations that the SNPs in these GWAS studies are the only SNPs that have been reported. Thus the total number of SNPs may be less than the amount reported above.

### Reclassification after doubling the sample size

In order to assess how much current estimates of risk will vary as a result of additional GWAS discoveries in the near future, we also explored how much reclassification would be observed for each phenotype using SNPs hypothetically discovered from a GWAS with double the sample size of the most recent GWAS studies. For each SNP from the recent GWAS, we calculated the power (p_j_) to observe said effect size in a single-stage study with double the sample size. We then predicted that the total number of SNPs we would observe with that effect size would equal:

$$ {p}_j{M}_{T,j} $$

Using the new set of predicted SNPs for each phenotype, we simulated the genotypes for the newly predicted set of SNPs for a sample of 100,000 individuals of European descent, assuming each new SNP was in a unique locus. For example, if for a specific SNP reported in the most recent two-stage GWAS, we expect there to be 10 total SNPs with the same effect size and we have a predicted power of 0.8 to detect the SNP in a double sample size study, we simulated eight SNPs of that effect size and minor allele frequency. In fact, we simulate slightly more by simulating the ceiling of p_j_M_T,j_. Reclassification was then assessed using the SNPs in the 2013 GWAS vs. when we had the sum of p_j_M_T,j_ for all SNPs. We then calculated the AUC in these simulated datasets. Since the AUC from the ‘current’ here is based just on the SNPs from a subset of all GWAS studies, it will not be directly comparable to the AUC calculated using the NHGRI catalog and the recent large scale GWAS.

## Results

**Fig. 1:**
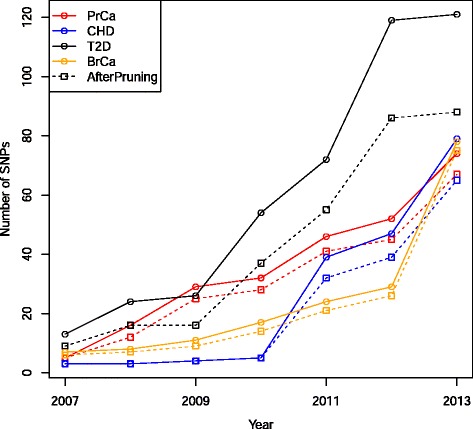
Total number of SNPs over a 2-year time period. The total number of SNPs reaching genome-wide significance with each disease increased over time. The dashed lines indicate the number of SNPs remaining after pruning out SNPs based on LD structure

**Fig. 2:**
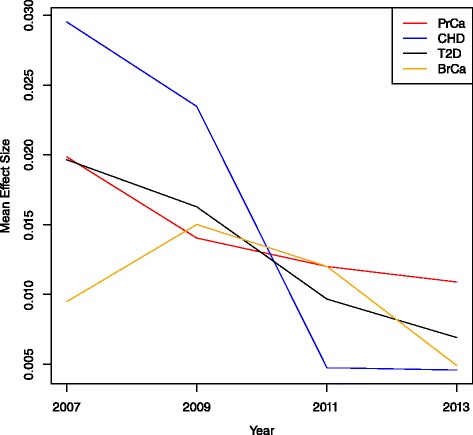
Mean SNP effect size by a 2-year time period. With larger sample sizes, GWAS were able to detect SNPs with smaller and smaller effect sizes. This brought down the mean effect size for each disease by year (with the exception of PrCa in 2009 which saw a slight bump)

**Fig. 3:**
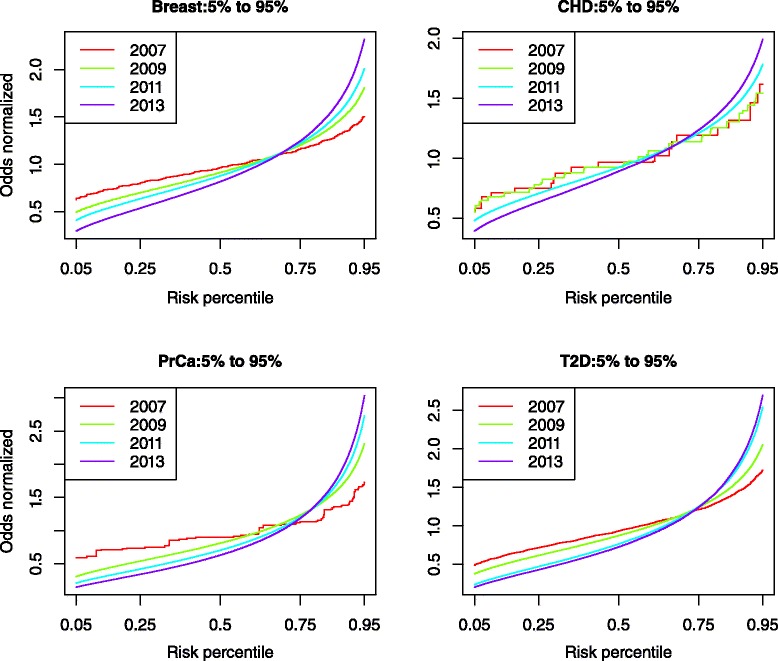
Risk distributions for each phenotype at 2007, 2009, 2011, and 2013. As more SNPs were discovered the distribution of cell type risks widened. This led to more individuals being placed in the tail ends of the distribution as time progressed

The number of SNPs meeting the selected *P* value and LD criteria outlined above increased steadily for all the phenotypes over successive time windows, with the most dramatic increase across all phenotypes occurring from 2011 to 2013 (Fig. 1). As the number of SNPs identified increased, the average effect size per SNP generally decreased (Fig. 2). As more SNPs were discovered, the proportion of individuals classified as Higher Risk also increased (Table 1) and the risk distribution for each phenotype widened (Fig. 3). The proportion of individuals that were reclassified increased substantially as more SNPs were discovered (Table 2). The highest 2-year reclassification rate for each phenotype occurred from 2011 to 2013, and the average reclassification rate from 2011 to 2013 across the phenotypes was 20.4 % and ranged from 16.3 % to 24.4 %. This was also the time period that saw the greatest increase in identified SNPs. Most of this reclassification reflected an improvement in the risk model, as indicated in the positive net reclassification indices in Table 3. Using the three risk categories (Lower, Average, and Higher), the majority of reclassification from 2011 to 2013 was due to movement between the Lower Risk and the Average Risk categories (see Additional file 1: Tables S1a, S2a, S3a, S4a). If the risk categories are grouped into Higher Risk versus Lower or Average Risk, the reclassification proportions from 2011 to 2013 are 7.0 %, 5.2 %, 5.5 %, and 7.4 % for BrCa, CHD, T2D, and PrCa, respectively. The full reclassification tables can be seen in Additional file 1: Tables S1–S4.

**Table 1:** Proportion of individuals at high risk (>2× average), by year

Disease	2007	2009	2011	2013
BrCa	0.002	0.029	0.051	0.079
CHD	0	0.003	0.026	0.049
T2D	0.020	0.055	0.094	0.103
PrCa	0.029	0.076	0.099	0.112

**Table 2:** Reclassification proportion based on year of SNP set

	BrCa	CHD	T2D	PrCa
2007 vs. 2009	0.083	0.003	0.126	0.269
2009 vs. 2011	0.131	0.091	0.38	0.224
2011 vs. 2013	0.237	0.163	0.172	0.244
2007 vs. 2013	0.295	0.177	0.426	0.504

Entries represent proportion of subjects whose genetic risk category (lower: <0.5× average; moderate: between 0.5× and 2.0× average; higher: >2× average) changes from one year to the next

**Table 3:** Net Reclassification Index based on year of SNP set

	BrCa	CHD	T2D	PrCa
2007 vs. 2009	0.067	0.004	0.095	0.211
2009 vs. 2011	0.075	0.065	0.254	0.131
2011 vs. 2013	0.137	0.089	0.061	0.121
2007 vs. 2013	0.274	0.146	0.389	0.510

The Net Reclassification Index is defined in the section ‘Reclassification and AUC’

We separately analyzed reclassification among those who were in the Higher Risk group in 2011 (Table 4). This was done to assess how much movement there was for individuals most likely to have changes in clinical management due to being classified at high risk. For BrCa, 42 % of the individuals who had been classified as high risk in 2011 moved to a lower risk classification in 2013. Reclassification proportions from 2011 to 2013 for the other three phenotypes ranged from 25 % to 55 %. The vast majority of this reclassification was from Higher Risk to Average Risk, with little movement from Higher Risk to Lower Risk (Additional file 1: Table S5).

**Table 4:** Proportion of higher risk (>2× average) individuals reclassified from Higher Risk to Average Risk or Lower Risk categories (<2× average)

	BrCa	CHD	T2D	PrCa
2007 vs. 2009	0.614	-------	0.04	0.378
2009 vs. 2011	0.325	0.622	0.661	0.248
2011 vs. 2013	0.418	0.550	0.249	0.309
2007 vs. 2013	0.586	-------	0.667	0.532

Dashes indicate that no individuals were classified as higher risk at one of the relevant time points

The AUC for the risk prediction models increased modestly from 2007 to 2013, with a mean increase over each 2-year period since 2007 of 0.034 across the four phenotypes. Two examples of larger increases include PrCa from 2007 to 2009 (0.591 - >0.667) and BrCa from 2011 to 2013 (0.634 - >0.671) (Table 5). This may be due to the large amount of SNPs that were discovered in the Michailidou *et al.* paper released in early 2013 [25]. For PrCa, there were 20 SNPs discovered from 2007 to 2009, bringing the number from five to 25 [27, 40]. The AUC values reported in Table 5 are generally slightly higher than those reported in the literature when validating risk models using similar number of SNPs [20, 41–55]. This is likely due to the compounded effect of overestimates in published individual-SNP ORs [56, 57].

**Table 5:** AUC based on year of SNP set

	BrCa	CHD	T2D	PrCa
Year 2007	0.574	0.58	0.606	0.596
Year 2009	0.612	0.582	0.642	0.671
Year 2011	0.636	0.611	0.695	0.717
Year 2013	0.672	0.636	0.712	0.748

When projecting the performance of risk prediction models based on hypothetical future GWAS with twice the sample size of the current largest studies, we observed marked reclassification, on the order of that observed comparing the model based on 2013 SNPs to that based on the 2007 SNPs: reclassification rates were 0.40, 0.12, 0.31, and 0.27 for BrCa, CHD, T2D and PrCa, respectively (Table 6). The net reclassification rates were again positive, indicating model improvement: net reclassification rates were 0.34, 0.082, 0.21, and 0.21 for BrCa, CHD, T2D, and PrCa, respectively (Table 6 and Additional file 1: Table S6). The proportion of individuals moving from Higher Risk to the Lower or Moderate Risk category remained large, ranging from 0.40 to 0.51 (Table 6). Individuals currently classified as Lower Risk or Average Risk were slightly less likely to be reclassified after doubling the sample size. For example, 30 % of individuals currently classified as Lower Risk for CHD were reclassified to Average Risk (none were reclassified as Higher Risk), while only 11 % of those currently classified as Average Risk were reclassified as Lower Risk or Higher Risk.

**Table 6:** Reclassification when sample size doubled

		Future SNPs			Future SNPs		
		BrCa			CHD		
Current SNPS	Risk	Low	Average	High	Low	Average	High
	Low	0.074	0.026	0.000	0.016	0.007	0
	Average	0.265	0.508	0.083	0.076	0.857	0.033
	High	0.001	0.021	0.021	0.000	0.004	0.006
		T2D			PrCa		
Current SNPS	Risk	Low	Average	High	Low	Average	High
	Low	0.120	0.037	0	0.040	0.016	0.000
	Average	0.182	0.535	0.065	0.178	0.676	0.066
	High	0.000	0.027	0.034	0.000	0.012	0.012

Entries are the proportion of individuals who are classified as Lower Risk (<0.5× average), Average Risk (between <0.5× average and >2× average), or Higher Risk (>2× average) risk based on: (1) currently known risk SNPs (rows) and (2) the risk SNPs known after a hypothetical future GWAS that doubles the size of the largest current GWAS (columns)

One limitation to using reclassification rates as a primary endpoint is that the degree of change in risk for individuals and the cohort as a whole is obscured. For example, one potential explanation for the reclassification rates we observed is that many individuals had small absolute changes in odds which nonetheless resulted in a change in risk categorization based on our defined odds thresholds. Across all phenotypes, roughly half of individuals (51 %) had a change in the adjusted odds of 0.2 or less from 2011 to 2013. However, 17 % of individuals had changes in adjusted odds of greater than 0.5.

## Discussion

Individuals’ genetic risk prediction for common complex diseases will change as our knowledge of the genetic underpinnings of these traits changes. At some point, as the known genetic contribution increases and approaches the total (known and unknown proportion) genetic contribution of common SNPs, the change in individuals’ predictions with every new discovery will diminish. We have shown that, depending on phenotype, between 18 % and 50 % of individual risk estimates were reclassified over the time period of 2007 to 2013. Rather than diminishing, the impact of new GWAS-discovered risk alleles remains high and may in fact be increasing, as studies reach a sample size ‘tipping point’ and have power to reliably detect many alleles with smaller effects. Eventually we will reach a point of diminishing returns, where larger and larger studies will fail to detect any novel SNPs, but for the time being we are still in the discovery stage.

The projected reclassification rate in the near future partly reflects the modest predictive power of current genetic risk prediction algorithms. It also reflects the fact that many common complex phenotypes appear to follow a polygenic inheritance model, with many SNPs of small effect contributing to disease risk. Previous GWAS have had low power to detect most of these SNPs, so published SNPs to date likely reflect only a portion of the heritability attributable to common genetic variation.

### Increasing numbers of individuals classified as Higher Risk and Lower Risk

We observed that the proportions of individuals in the Higher Risk category, defined as greater than 2× the mean risk, increased over time*.* Specifically*,* we noted a gradual increase in the proportion of individuals at greater than twice the average genetic risk from less than 3 % in 2007 to a total of 5 % to 11 % in 2013 across the four phenotypes. Additionally, increased proportions in the Higher Risk category were noted when risk estimates were projected using double the GWAS sample size. This result was anticipated given that as more risk-associated SNPs are discovered, the total risk distribution widens, and therefore the proportion of individuals at increased risk increases slightly. In other words, discovering new SNPs increases the probability of discovering individuals at the extremes of genetic risk burden.

Genetic risk assessment for a given multifactorial condition is most relevant for those who fall in the Higher Risk or Lower Risk categories. In theory, as certainty around risk estimates improve, early or enhanced screening protocols and stricter control of risk factors through lifestyle modification or medical intervention may be warranted for individuals with increased genetic risk profiles. On the other hand, individuals with relatively low genetic risk factors may benefit or avoid disadvantage if they are subject to less screening or fewer preventative measures. For the vast majority with average genetic risk, little change would appear warranted from conventional screening and prevention measures. Currently and in the near future, however, the emphasis is and will be likely placed on patients with increased risk. As individuals in the extremes of the predicted risk distribution are the most important to identify from a clinical decision-making perspective, the possibility of identifying more such individuals over time offers a reason to suggest that risk classification based on common variation may have increased clinical utility in the future.

### Potential clinical implications of high reclassification rates

There are pressing questions concerning the validity of risk estimates for those interested in translating the current discoveries from the GWAS era into clinical recommendations, and this study is not intended to address these issues. Our analysis does not address or attempt to account for non-genetic components of risk estimation for any phenotype, such as dietary factors, smoking, or other exposures. Non-genetic risk factors are important components of risk, and for some phenotypes, contribute more to risk of developing disease than does genetic variation. In clinical assessment, therefore, evaluation of non-genetic risk factors will remain important even as risk-prediction models based on common genetic variation improve. Our study, however, focuses only on the genetic risk and how the rapidly evolving data about common genetic variation impacts risk categorization over time. Moreover, we have not addressed the important issue of risk model calibration—whether those predicted to be at high risk are indeed at high risk. Model calibration requires large genotyped cohorts with extensive follow-up, and is beyond the scope of our analyses.

A key component of the validity of risk projections based on common genetic variation, however, is whether risk predictions are stable as additional SNPs are identified. In other words, what proportion of individuals from the clinically important subgroup currently classified as Higher Risk remains in the Higher Risk category as more SNPs are discovered for a given phenotype? If reclassification is low for those in the Higher Risk category, then considering modifications to screening or preventative measures may be warranted based on current risk estimates. However, if a large proportion of individuals identified as Higher Risk based on early models were later classified as Average Risk or Lower Risk, additional caution would be warranted before basing clinical decisions on a predicted increased risk for a phenotype.

Our analysis, based only on the identification of new risk alleles, shows a significant proportion of reclassification for individuals determined to be in the Higher Risk category for each phenotype for the 2-year period as recent as 2011 to 2013. For an individual who underwent a risk evaluation based on known risk-associated SNPs for CHD in 2011, there was a 55 % probability that they would subsequently be reclassified into a lower risk group in 2013 and therefore may have received misguided intervention. Though the downward reclassification rates are lower for other phenotypes, even the smallest rate is 25 % for T2D. Projections based on doubling the GWAS sample size suggest that even much larger samples than are currently available do not resolve the ongoing instability, with projected reclassification rates for individuals at high risk near 50 % for all four phenotypes.

We stress that the changes in reclassification rates and net reclassification indices over time that we report here are for illustration purposes only. Both reclassification rates and net reclassification indices are sensitive to the choice of risk thresholds used [58]. The ultimate criteria for benchmarking prediction models against each other depend on the specifics of the clinical setting (including the existence of agreed-upon risk thresholds, specific interventions, and costs and benefits of these interventions) [59, 60]. Precisely because such important evaluations are so context dependent, we have not undertaken them here.

## Conclusions

In sum, our analysis shows significant instability in predicted risk from common genetic variation for four multifactorial phenotypes. While the results may not be unexpected given the known limitations of the risk prediction using genetic data, they underscore the caution that should be used in interpreting results for an individual patient. Moreover, this analysis demonstrates a consequence of the limited predictive ability of current genetic models, specifically increased reclassification rates in the face of new data. At the same time, reclassification rates should eventually begin to decrease as additional SNPs are identified and increasing proportions of the general population will fall into the clinically relevant Lower Risk and Higher Risk categories, resulting in the opportunity for potentially impactful interventions particularly for individuals in the Higher Risk group. Moreover, the major improvements in number of known SNPs in our analysis underscore the progress that has been and will be made. Hope for potential clinical utility is warranted even in the face of the significant unresolved limitations and challenges.

## Additional file

Additional file 1:
**The following additional data are available with the online version of this paper.** **Table S1a.** Reclassification results for BrCa. **Table S1b.** Reclassification in cases for BrCa. **Table S2aa** Reclassification results for CHD. **Table S2b.** Reclassification in cases for CHD. **Table S3a.** Reclassification results for T2D. **Table S3b.** Reclassification in cases for T2D. **Table S4a.** Reclassification results for PrCa. **Table S4b.** Reclassification for cases for PrCa. **Table S5.** Proportion of Higher Risk individuals reclassified from Higher Risk to Lower Risk categories. **Table S6.** Reclassification in cases when sample size doubled. (DOCX 24 kb)

## References

[1] Bowles Biesecker B, Marteau TM (1999). The future of genetic counselling: an international perspective. Nat Genet.

[2] Yang Q, Khoury MJ, Botto L, Friedman JM, Flanders WD (2003). Improving the prediction of complex diseases by testing for multiple disease-susceptibility genes. Am J Hum Genet.

[3] Guttmacher AE, Collins FS (2002). Genomic medicine--a primer. N Engl J Med.

[4] Weedon MN, McCarthy MI, Hitman G, Walker M, Groves CJ, Zeggini E (2006). Combining information from common type 2 diabetes risk polymorphisms improves disease prediction. PLoS Med.

[5] Collins FS (1999). Shattuck lecture--medical and societal consequences of the Human Genome Project. N Engl J Med.

[6] Holtzman NA, Marteau TM (2000). Will genetics revolutionize medicine?. N Engl J Med.

[7] Kolor K, Duquette D, Zlot A, Foland J, Anderson B, Giles R (2012). Public awareness and use of direct-to-consumer personal genomic tests from four state population-based surveys, and implications for clinical and public health practice. Genet Med.

[8] Frueh FW, Greely HT, Green RC, Hogarth S, Siegel S (2011). The future of direct-to-consumer clinical genetic tests. Nature reviews. Genetics.

[9] Manolio TA (2010). Genomewide association studies and assessment of the risk of disease. N Engl J Med.

[10] Kalf RR, Mihaescu R, Kundu S, de Knijff P, Green RC, Janssens AC (2014). Variations in predicted risks in personal genome testing for common complex diseases. Genet Med.

[11] Bloss CS, Wineinger NE, Darst BF, Schork NJ, Topol EJ (2013). Impact of direct-to-consumer genomic testing at long term follow-up. J Med Genet.

[12] Gallagher PKJ, Carrere AD, Chen C, Cupples LA, Roberts JS, Green RC, PGEN Study Group. Healthcare Utilization Following Personal Genomic Testing. Paper presented at: American College of Medical Genetics and Genomics Annual Meeting; March 27, 2015, 2015; Salt Lake City, UT.

[13] Gutierrez A. FDA Warning Letter to 23andMe. 2013; http://www.fda.gov/ICECI/EnforcementActions/WarningLetters/2013/ucm376296.htm. Accessed 11 Jan 2014.

[14] Green RC, Farahany NA (2014). Regulation: The FDA is overcautious on consumer genomics. Nature.

[15] Biesecker LG, Green RC (2014). Diagnostic clinical genome and exome sequencing. N Engl J Med.

[16] Dewey FE, Grove ME, Pan C, Goldstein BA, Bernstein JA, Chaib H (2014). Clinical interpretation and implications of whole-genome sequencing. JAMA.

[17] Ashley EA, Butte AJ, Wheeler MT, Chen R, Klein TE, Dewey FE (2010). Clinical assessment incorporating a personal genome. Lancet.

[18] Vassy JL, Lautenbach DM, McLaughlin HM, Kong SW, Christensen KD, Krier J (2014). The MedSeq Project: a randomized trial of integrating whole genome sequencing into clinical medicine. Trials.

[19] Kong SW, Lee IH, Leshchiner I, Krier J, Kraft P, Rehm HL (2015). Summarizing polygenic risks for complex diseases in a clinical whole-genome report. Genet Med.

[20] Aschard H, Chen J, Cornelis MC, Chibnik LB, Karlson EW, Kraft P (2012). Inclusion of gene-gene and gene-environment interactions unlikely to dramatically improve risk prediction for complex diseases. Am J Hum Genet.

[21] Mihaescu R, van Hoek M, Sijbrands EJ, Uitterlinden AG, Witteman JC, Hofman A (2009). Evaluation of risk prediction updates from commercial genome-wide scans. Genet Med.

[22] Hindorff LA, Sethupathy P, Junkins HA, Ramos EM, Mehta JP, Collins FS (2009). Potential etiologic and functional implications of genome-wide association loci for human diseases and traits. Proc Natl Acad Sci U S A.

[23] Macpherson M NB, Hsu A, Mountain J. White paper 23-01: estimating genotype-specific incidence for one or several loci. 2007; https://23andme.https.internapcdn.net/res/pdf/HIC-SXIYiYqXreldAxO5yA_23-01_Estimating_Genotype_Specific_Incidence.pdf. Accessed 24 April 2014.

[24] Consortium IS, Purcell SM, Wray NR, Stone JL, Visscher PM, O’Donovan MC (2009). Common polygenic variation contributes to risk of schizophrenia and bipolar disorder. Nature.

[25] Michailidou K, Hall P, Gonzalez-Neira A, Ghoussaini M, Dennis J, Milne RL (2013). Large-scale genotyping identifies 41 new loci associated with breast cancer risk. Nat Genet.

[26] Morris AP, Voight BF, Teslovich TM, Ferreira T, Segre AV, Steinthorsdottir V (2012). Large-scale association analysis provides insights into the genetic architecture and pathophysiology of type 2 diabetes. Nat Genet.

[27] Eeles RA, Olama AA, Benlloch S, Saunders EJ, Leongamornlert DA, Tymrakiewicz M (2013). Identification of 23 new prostate cancer susceptibility loci using the iCOGS custom genotyping array. Nat Genet.

[28] Deloukas P, Kanoni S, Willenborg C, Farrall M, Assimes TL, Thompson JR (2013). Large-scale association analysis identifies new risk loci for coronary artery disease. Nat Genet.

[29] Johnson AD, Handsaker RE, Pulit SL, Nizzari MM, O’Donnell CJ, de Bakker PI (2008). SNAP: a web-based tool for identification and annotation of proxy SNPs using HapMap. Bioinformatics.

[30] International HapMap Consortium (2003). The International HapMap Project. Nature.

[31] Abecasis GR, Altshuler D, Auton A, Brooks LD, Durbin RM, 1000 Genomes Project Consortium (2010). A map of human genome variation from population-scale sequencing. Nature.

[32] DJ SJaS. haplo.stats: Statistical Analysis of Haplotypes with Traits and Covariates when Linkage Phase is Ambiguous. R package version 1.4.4. 2009; http://CRAN.R-project.org/package=haplo.stats.

[33] Saccone SF, Quan J, Jones PL (2012). BioQ: tracing experimental origins in public genomic databases using a novel data provenance model. Bioinformatics.

[34] Cook NR (2007). Use and misuse of the receiver operating characteristic curve in risk prediction. Circulation.

[35] Roberts NJ, Vogelstein JT, Parmigiani G, Kinzler KW, Vogelstein B, Velculescu VE (2012). The predictive capacity of personal genome sequencing. Sci Trans Med.

[36] Pepe MS (2003). The statistical evaluation of medical tests for classification and prediction.

[37] Park JH, Wacholder S, Gail MH, Peters U, Jacobs KB, Chanock SJ (2010). Estimation of effect size distribution from genome-wide association studies and implications for future discoveries. Nat Genet.

[38] Skol AD, Scott LJ, Abecasis GR, Boehnke M (2006). Joint analysis is more efficient than replication-based analysis for two-stage genome-wide association studies. Nat Genet.

[39] Schumacher FR, Berndt SI, Siddiq A, Jacobs KB, Wang Z, Lindstrom S (2011). Genome-wide association study identifies new prostate cancer susceptibility loci. Hum Mol Genet.

[40] Gudmundsson J, Sulem P, Gudbjartsson DF, Blondal T, Gylfason A, Agnarsson BA (2009). Genome-wide association and replication studies identify four variants associated with prostate cancer susceptibility. Nat Genet.

[41] Lyssenko V, Laakso M (2013). Genetic screening for the risk of type 2 diabetes: worthless or valuable?. Diabetes Care.

[42] Bao W, Hu FB, Rong S, Rong Y, Bowers K, Schisterman EF (2013). Predicting risk of type 2 diabetes mellitus with genetic risk models on the basis of established genome-wide association markers: a systematic review. Am J Epidemiol.

[43] Walford GA, Porneala BC, Dauriz M, Vassy JL, Cheng S, Rhee EP (2014). Metabolite traits and genetic risk provide complementary information for the prediction of future type 2 diabetes. Diabetes Care.

[44] Chatterjee N, Wheeler B, Sampson J, Hartge P, Chanock SJ, Park JH (2013). Projecting the performance of risk prediction based on polygenic analyses of genome-wide association studies. Nat Genet.

[45] Do CB, Hinds DA, Francke U, Eriksson N (2012). Comparison of family history and SNPs for predicting risk of complex disease. PLoS Genet.

[46] Vaarhorst AA, Lu Y, Heijmans BT, Dolle ME, Bohringer S, Putter H (2012). Literature-based genetic risk scores for coronary heart disease: the Cardiovascular Registry Maastricht (CAREMA) prospective cohort study. Circ Cardiovasc Genet.

[47] Mavaddat N, Pharoah PD, Michailidou K, Tyrer J, Brook MN, Bolla MK (2015). Prediction of breast cancer risk based on profiling with common genetic variants. J Natl Cancer Inst.

[48] Aschard H, Zaitlen N, Lindstrom S, Kraft P (2015). Variation in predictive ability of common genetic variants by established strata: the example of breast cancer and age. Epidemiology.

[49] Husing A, Canzian F, Beckmann L, Garcia-Closas M, Diver WR, Thun MJ (2012). Prediction of breast cancer risk by genetic risk factors, overall and by hormone receptor status. J Med Genet.

[50] Darabi H, Czene K, Zhao W, Liu J, Hall P, Humphreys K (2012). Breast cancer risk prediction and individualised screening based on common genetic variation and breast density measurement. Breast Cancer Res.

[51] van Zitteren M, van der Net JB, Kundu S, Freedman AN, van Duijn CM, Janssens AC (2011). Genome-based prediction of breast cancer risk in the general population: a modeling study based on meta-analyses of genetic associations. Cancer Epidemiol Biomarkers Prev.

[52] Szulkin R, Whitington T, Eklund M, Aly M, Eeles RA, Easton D (2015). Prediction of individual genetic risk to prostate cancer using a polygenic score. Prostate.

[53] Butoescu V, Ambroise J, Stainier A, Dekairelle AF, Gala JL, Tombal B (2014). Does genotyping of risk-associated single nucleotide polymorphisms improve patient selection for prostate biopsy when combined with a prostate cancer risk calculator?. Prostate.

[54] Johansson M, Holmstrom B, Hinchliffe SR, Bergh A, Stenman UH, Hallmans G (2012). Combining 33 genetic variants with prostate-specific antigen for prediction of prostate cancer: longitudinal study. Int J Cancer.

[55] Lindstrom S, Schumacher FR, Cox D, Travis RC, Albanes D, Allen NE (2012). Common genetic variants in prostate cancer risk prediction--results from the NCI Breast and Prostate Cancer Cohort Consortium (BPC3). Cancer Epidemiol Biomarkers Prev.

[56] Ioannidis JP (2008). Why most discovered true associations are inflated. Epidemiology.

[57] Kraft P (2008). Curses—winner’s and otherwise--in genetic epidemiology. Epidemiology.

[58] Muhlenbruch K, Heraclides A, Steyerberg EW, Joost HG, Boeing H, Schulze MB (2013). Assessing improvement in disease prediction using net reclassification improvement: impact of risk cut-offs and number of risk categories. Eur J Epidemiol.

[59] Kerr KF, Bansal A, Pepe MS (2012). Further insight into the incremental value of new markers: the interpretation of performance measures and the importance of clinical context. Am J Epidemiol.

[60] Pepe MS (2011). Problems with risk reclassification methods for evaluating prediction models. Am J Epidemiol.

