# Supplementary material for: Reclassification of genetic-based risk predictions as GWAS data accumulate
Source: Genome Med. 2016 Feb 17;8:20. doi: 10.1186/s13073-016-0272-5 (PMC4756503; doi:10.1186/s13073-016-0272-5)
Supplement: Additional file 1: — The following additional data are available with the online version of this paper. Table S1a. Reclassification results for BrCa. Table S1b. Reclassification in cases for BrCa. Table S2aa Reclassification results for CHD. Table S2b. Reclassification in cases for CHD. Table S3a. Reclassification results for T2D. Table S3b. Reclassification in cases for T2D. Table S4a. Reclassification results for PrCa. Table S4b. Reclassification for cases for PrCa. Table S5. Proportion of Higher Risk individuals reclassified from Higher Risk to Lower Risk categories. Table S6. Reclassification in cases when sample size doubled. (DOCX 24 kb) [file 13073_2016_272_MOESM1_ESM.docx]

**Additional file 1**

**Table S1a: Reclassification in the general population / controls for BrCa**

|  | **2007 vs. 2009** | **2009 vs. 2011** | **2011 vs. 2013** | **2007 vs. 2013** |
| --- | --- | --- | --- | --- |
| **Lower to Lower** | 0.00110 | 0.03999 | 0.08296 | 0.00125 |
| **Lower To Average** | 0.00064 | 0.01350 | 0.03336 | 0.00049 |
| **Lower to Higher** | 0.00000 | 0.00000 | 0.00002 | 0.00000 |
| **Average to Lower** | 0.05239 | 0.07635 | 0.13347 | 0.21524 |
| **Average to Average** | 0.91521 | 0.80950 | 0.65011 | 0.70304 |
| **Average to Higher** | 0.02851 | 0.03132 | 0.04896 | 0.07783 |
| **Higher to Lower** | 0.00000 | 0.00000 | 0.00008 | 0.00002 |
| **Higher to Average** | 0.00132 | 0.00954 | 0.02130 | 0.00124 |
| **Higher to Higher** | 0.00083 | 0.01980 | 0.02974 | 0.00089 |

**Table S1b: Reclassification in cases for BrCa**

|  | **2007 vs. 2009** | **2009 vs. 2011** | **2011 vs. 2013** | **2007 vs. 2013** |
| --- | --- | --- | --- | --- |
| **Lower to Lower** | 0.00042 | 0.01485 | 0.02735 | 0.00041 |
| **Lower To Average** | 0.00039 | 0.00816 | 0.02246 | 0.00037 |
| **Lower to Higher** | 0.00000 | 0.00000 | 0.00004 | 0.00000 |
| **Average to Lower** | 0.02314 | 0.03278 | 0.05174 | 0.07871 |
| **Average to Average** | 0.90445 | 0.79931 | 0.64850 | 0.70020 |
| **Average to Higher** | 0.06733 | 0.07434 | 0.12513 | 0.21598 |
| **Higher to Lower** | 0.00000 | 0.00000 | 0.00000 | 0.00000 |
| **Higher to Average** | 0.00210 | 0.01596 | 0.03118 | 0.00158 |
| **Higher to Higher** | 0.00218 | 0.05461 | 0.09355 | 0.00275 |

**Table S2a: Reclassification in the general population / controls for CHD**

|  | **2007 vs. 2009** | **2009 vs. 2011** | **2011 vs. 2013** | **2007 vs. 2013** |
| --- | --- | --- | --- | --- |
| **Lower to Lower** | 0.00632 | 0.00223 | 0.03728 | 0.00165 |
| **Lower To Average** | 0.00005 | 0.00409 | 0.02435 | 0.00469 |
| **Lower to Higher** | 0.00000 | 0.00000 | 0.00001 | 0.00003 |
| **Average to Lower** | 0.00000 | 0.05941 | 0.08718 | 0.12281 |
| **Average to Average** | 0.99030 | 0.90597 | 0.78763 | 0.82172 |
| **Average to Higher** | 0.00333 | 0.02497 | 0.03732 | 0.04910 |
| **Higher to Lower** | 0.00000 | 0.00000 | 0.00000 | 0.00000 |
| **Higher to Average** | 0.00000 | 0.00207 | 0.01443 | 0.00000 |
| **Higher to Higher** | 0.00000 | 0.00126 | 0.01180 | 0.00000 |

**Table S2b: Reclassification in cases for CHD**

|  | **2007 vs. 2009** | **2009 vs. 2011** | **2011 vs. 2013** | **2007 vs. 2013** |
| --- | --- | --- | --- | --- |
| **Lower to Lower** | 0.00270 | 0.00089 | 0.01363 | 0.00064 |
| **Lower To Average** | 0.00003 | 0.00281 | 0.01631 | 0.00387 |
| **Lower to Higher** | 0.00000 | 0.00000 | 0.00002 | 0.00007 |
| **Average to Lower** | 0.00000 | 0.02533 | 0.03617 | 0.04916 |
| **Average to Average** | 0.99004 | 0.90649 | 0.78984 | 0.82392 |
| **Average to Higher** | 0.00723 | 0.05811 | 0.09032 | 0.12233 |
| **Higher to Lower** | 0.00000 | 0.00000 | 0.00000 | 0.00000 |
| **Higher to Average** | 0.00000 | 0.00308 | 0.02164 | 0.00000 |
| **Higher to Higher** | 0.00000 | 0.00328 | 0.03206 | 0.00000 |

**Table S3a Reclassification in the general population / controls for T2D**

|  | **2007 vs. 2009** | **2009 vs. 2011** | **2011 vs. 2013** | **2007 vs. 2013** |
| --- | --- | --- | --- | --- |
| **Lower to Lower** | 0.05130 | 0.07228 | 0.23706 | 0.02478 |
| **Lower To Average** | 0.00148 | 0.06470 | 0.03863 | 0.02650 |
| **Lower to Higher** | 0.00000 | 0.00194 | 0.00000 | 0.00150 |
| **Average to Lower** | 0.08762 | 0.20284 | 0.07813 | 0.28969 |
| **Average to Average** | 0.80339 | 0.52932 | 0.52015 | 0.54297 |
| **Average to Higher** | 0.03597 | 0.07351 | 0.03180 | 0.09432 |
| **Higher to Lower** | 0.00000 | 0.00057 | 0.00000 | 0.00072 |
| **Higher to Average** | 0.00080 | 0.03606 | 0.02348 | 0.01279 |
| **Higher to Higher** | 0.01944 | 0.01878 | 0.07075 | 0.00673 |

**Table S3b: Reclassification in cases for T2D**

|  | **2007 vs. 2009** | **2009 vs. 2011** | **2011 vs. 2013** | **2007 vs. 2013** |
| --- | --- | --- | --- | --- |
| **Lower to Lower** | 0.01720 | 0.02239 | 0.07046 | 0.00764 |
| **Lower To Average** | 0.00080 | 0.05541 | 0.02365 | 0.02375 |
| **Lower to Higher** | 0.00000 | 0.00490 | 0.00000 | 0.00426 |
| **Average to Lower** | 0.03796 | 0.07161 | 0.03240 | 0.09493 |
| **Average to Average** | 0.80325 | 0.52681 | 0.51793 | 0.54064 |
| **Average to Higher** | 0.08269 | 0.21249 | 0.07691 | 0.29069 |
| **Higher to Lower** | 0.00000 | 0.00000 | 0.00000 | 0.00000 |
| **Higher to Average** | 0.00148 | 0.04583 | 0.03829 | 0.01549 |
| **Higher to Higher** | 0.05663 | 0.06033 | 0.24035 | 0.02231 |

**Table S4a: Reclassification in the general population / controls for PrCa**

|  | **2007 vs. 2009** | **2009 vs. 2011** | **2011 vs. 2013** | **2007 vs. 2013** |
| --- | --- | --- | --- | --- |
| **Lower to Lower** | 0.01243 | 0.18504 | 0.27795 | 0.01232 |
| **Lower To Average** | 0.00389 | 0.02343 | 0.04770 | 0.00378 |
| **Lower to Higher** | 0.00000 | 0.00001 | 0.00004 | 0.00022 |
| **Average to Lower** | 0.19605 | 0.14065 | 0.12176 | 0.38610 |
| **Average to Average** | 0.70078 | 0.53346 | 0.41008 | 0.47038 |
| **Average to Higher** | 0.05796 | 0.04144 | 0.04392 | 0.09831 |
| **Higher to Lower** | 0.00000 | 0.00000 | 0.00000 | 0.00129 |
| **Higher to Average** | 0.01088 | 0.01887 | 0.03045 | 0.01407 |
| **Higher to Higher** | 0.01801 | 0.05710 | 0.06810 | 0.01353 |

**Table S4b: Reclassification in cases for PrCa**

|  | **2007 vs. 2009** | **2009 vs. 2011** | **2011 vs. 2013** | **2007 vs. 2013** |
| --- | --- | --- | --- | --- |
| **Lower to Lower** | 0.00375 | 0.05237 | 0.07155 | 0.00284 |
| **Lower To Average** | 0.00280 | 0.01471 | 0.03275 | 0.00331 |
| **Lower to Higher** | 0.00000 | 0.00002 | 0.00009 | 0.00066 |
| **Average to Lower** | 0.07406 | 0.05509 | 0.04638 | 0.11459 |
| **Average to Average** | 0.68717 | 0.52113 | 0.40554 | 0.46342 |
| **Average to Higher** | 0.15349 | 0.10672 | 0.11925 | 0.33913 |
| **Higher to Lower** | 0.00000 | 0.00000 | 0.00000 | 0.00000 |
| **Higher to Average** | 0.01617 | 0.02990 | 0.04532 | 0.01688 |
| **Higher to Higher** | 0.06256 | 0.22006 | 0.27913 | 0.05867 |

**Table S5: Proportion of “Higher Risk” individuals reclassified from “Higher Risk” to “Lower Risk” category**

|  | **BrCa** | **CHD** | **T2D** | **PrCa** |
| --- | --- | --- | --- | --- |
| **2007 vs. 2009** | 0 | ------- | 0 | 0 |
| **2009 vs. 2011** | 0 | 0 | 0.010286952 | 0 |
| **2011 vs. 2013** | 0.001564945 | 0 | 0 | 0 |
| **2007 vs. 2013** | 0.009302326 | ------- | 0.035573123 | 0.044652129 |
| *Dashes indicate that no individuals were classified as higher risk at one of the relevant time points*. | | | | |

**Table S6. Reclassification in cases when sample size doubled**

|  |  | **BrCa** | | | **CHD** | | |
| --- | --- | --- | --- | --- | --- | --- | --- |
| **Current SNPS** | **Risk** | Low | Avg | High | Low | Avg | High |
|  | Low | 0.019 | 0.021 | 0.001 | 0.006 | 0.005 | 0.000 |
|  | Avg | 0.087 | 0.508 | 0.256 | 0.032 | 0.857 | 0.077 |
|  | High | 0.000 | 0.026 | 0.081 | 0 | 0.007 | 0.016 |
|  |  | **T2D**  **PrCa**  **T2D** | | | **PrCa**  **T2D**  **PrCa** | | |
| **Current SNPS** | **Risk** | Low | Avg | High | Low | Avg | High |
|  | Low | 0.034 | 0.027 | 0.001 | 0.012 | 0.011 | 0.000 |
|  | Avg | 0.066 | 0.533 | 0.179 | 0.067 | 0.675 | 0.178 |
|  | High | 0.000 | 0.037 | 0.124 | 0 | 0.016 | 0.040 |
